# Exploration of a pain assessment tool on burning mouth syndrome

**DOI:** 10.22514/jofph.2025.060

**Published:** 2025-09-12

**Authors:** Takumi Shimura, Tatsuki Itagaki, Ken-ichiro Sakata, Takuya Asaka, Masayuki Shinohara, Sadasuke Hayata, Ikuya Miyamoto

**Affiliations:** ^1^Oral Diagnosis and Medicine, Faculty of Dental Medicine, Hokkaido University, 060-8586 Sapporo, Japan

**Keywords:** Burning mouth syndrome (BMS), The short form of the McGill pain questionnaire version 2 (SF-MPQ-2), Oral dysesthesia, Research diagnostic criteria

## Abstract

**Background**: Burning mouth syndrome (BMS) is a chronic orofacial pain disorder. The etiology and pathophysiology of BMS remain unclear; multiple factors may interact in complex ways. There is a need for simpler and more cost-effective BMS evaluation criteria. This study aimed to evaluate the reliability and validity of the Short Form McGill Pain Questionnaire version 2 (SF-MPQ-2) in patients with BMS and develop a subscale based on factor analysis of the results to classify patients per their symptoms. **Methods**: Several factors such as patient characteristics (age, sex, smoking habit, and medical history), the SF-MPQ-2 (original: eleven-point rating scale and modified: four-point rating scale), and the numerical rating scale (NRS) of BMS were examined and analyzed. **Results**: In total, 38 patients were enrolled. Cronbach’s alpha was 0.93 (0.88–0.96) and 0.83 (0.74–0.90) for the SF-MPQ-2 (original) and SF-MPQ-2 (Modified), respectively. Only the correlation between the NRS and the SF-MPQ-2 (Modified) reached statistical significance. These results showed that the SF-MPQ-2 (Modified) were more reliable than the SF-MPQ-2 (Original). Factor analysis led to classification into three new factors. **Conclusions**: SF-MPQ-2 was useful for BMS. In current clinical practice, the modified questionnaire may yield similar or better results, and a more precise treatment strategy can be pursued by classifying responses according to the proposed subscales and examining treatment effects.

## 1. Introduction

Burning mouth syndrome (BMS) is defined as “idiopathic orofacial pain with intraoral burning or dysesthesia recurring daily for more than 2 hours per day and more than 3 months, without any identifiable causative lesions, with or without somatosensory changes” [1, 2]. BMS is recognized as a psychogenic disorder owing to its frequent association with anxiety and depressive symptoms [3, 4, 5, 6, 7]. Several studies have proposed research diagnostic criteria and emphasized the remarkable role of psychological factors in BMS [8]. Many pharmacological treatments for BMS include drugs frequently used to treat neuropathic pain [9]. However, the etiology and pathophysiology of BMS remain unclear; multiple factors can interact in complex ways [2, 3, 5, 7, 9].

In recent years, numerous studies have been conducted to identify biomarkers for the diagnosis and prognosis or assessment of BMS; however, these studies on biomarkers are costly and time-consuming [10, 11, 12, 13]. There are no biomarkers available for the assessment and diagnosis of burning mouth syndrome [10, 11, 12, 13]. Pain is highly subjective, but objective assessment remains a challenge. Studies on BMS have reported quantitative assessments in a single dimension using the visual analog scale and numerical rating scale (NRS); however, few studies have conducted qualitative and objective assessments [7, 14]. Therefore, easy-to-use assessment instruments are desirable and necessary [8].

The Short Form McGill Pain Questionnaire-2 (SF-MPQ-2) is a self-administered questionnaire that comprehensively assesses pain by incorporating neuropathic pain items into the original SF-MPQ. The SF-MPQ was developed as a multidimensional pain assessment tool and later modified to make its usage easier [15, 16, 17]. Pain type is divided into four subscales: continuous pain descriptors, intermittent pain descriptors, neuropathic pain descriptors, and affective descriptors. The 22 questionnaire’s items can assess pain intensity and its qualitative aspects, providing a comprehensive assessment of complex pain conditions [15]. The SF-MPQ-2 has been translated into several languages worldwide and validated in various settings. In particular, the Japanese version has demonstrated high reliability and validity for the assessment of general chronic pain [18, 19]. The SF-MPQ-2 has been used to assess general chronic pain, but the reliability and validity of the questionnaire needs to be verified for each disease [15]. No studies have assessed the reliability and validity of the SF-MPQ-2 for BMS.

This study aimed to assess the reliability and validity of the SF-MPQ-2 in patients with BMS and develop a subscale based on exploratory factor analysis to classify patients based on their symptoms.

## 2. Materials and methods

### 2.1 Patient selection

Patients were examined and diagnosed in our department based on the International Classification of Orofacial Pain (ICOP) 1st Edition Criteria [1, 8]. The diagnostic research criteria for BMS were based on the Currie *et al.*’s [8] proposal. The diagnostic protocol for BMS at the Department of Oral Diagnosis and Medicine, Hokkaido University is shown in Fig. 1. Because BMS is a diagnosis of exclusion, patients were not diagnosed with BMS if systemic and oral structural disorders were identified, which could account for oral pain. In other words, patients with an underlying medical condition, based on a clinical interview, or abnormalities detected by our diagnostic algorithm were excluded from the diagnosis of BMS. However, patients with residual symptoms after antifungal or replacement therapy for deficiency factors, such as trace metals and vitamins, were diagnosed at that time with BMS if the blood test results were within normal ranges. Since patients with conditions potentially related to oral pain were excluded, some patients with BMS and coexisting systemic conditions may not have been represented in the study.

**Fig. 1.** Diagnostic protocol for burning mouth syndrome at the department of oral diagnosis and medicine, Hokkaido University. BMS: Burning mouth syndrome.

Inclusion and Exclusion Criteria: Patients were seen at the Department of Diagnosis and Medicine at Hokkaido University Hospital between July and October 2024. Herein, 42 women patients with BMS with clear medical records of their treatment were enrolled. Exclusion criteria included patients with cognitive impairment, such as those unable to hold a conversation or communicate and with incomplete questionnaire or instrument data. Of 42 patients, 4 were excluded per the exclusion criteria. Finally, 38 patients were enrolled in the study. In Japan, oral medicine has been established as a subspecialty of oral surgery. All patients were diagnosed by a board-certified oral and maxillofacial surgeon with >10 years of clinical experience and accreditation by the Japanese Society of Oral and Maxillofacial Surgeons (KiS).

### 2.2 Procedure

The SF-MPQ-2 questionnaire was prepared in two formats: the original 11-point assessment form and the modified 4-point scale form (**Supplementary material**). Patients with BMS were surveyed before and after the consultation, with the order of the two questionnaires randomized. Questions to the dentist were not allowed during the survey, and no recommendations or omissions were noted. NRS was assessed simultaneously.

### 2.3 Study variables

Several factors such as patient characteristics (age, sex, smoking habits, and medical history), the SF-MPQ-2 (Original: eleven-point rating scale and Modified: four-point rating scale), and the NRS of BMS were examined. Smoking habit was defined as “yes” for patients who had a smoking habit the past 5 years.

### 2.4 Statistical analysis

Continuous data were presented as mean (standard deviation (SD)). The sensitivity to detect variation at the extreme ends of the scoring range were assessed by the floor and ceiling effects. The ceiling and floor effects were determined when the mean ± 1 SD exceeded the maximum and minimum measured values, respectively [9]. The reliability of each scale was assessed using Cronbach’s alpha. The concordance of SF-MPQ-2 was assessed by calculating the Pearson’s correlation coefficient between the total scores of the SF-MPQ-2 (original) and SF-MPQ-2 (Modified) due to the different rating scales. The concurrent validity was assessed by calculating the Pearson’s correlation coefficient between the total scores of the NRS and SF-MPQ-2 (original) or SF-MPQ-2 (Modified).

A scale of 0–3 was adopted to factor analysis because having 0–10 options makes it difficult for people to make a choice. The number of common factors was set at three before the exploratory factor analysis based on the parallel analysis. Factor loadings were calculated using maximum likelihood estimation, and factor axes were rotated using varimax rotation. Factor loadings of ≥0.4 were used to define subscales. The internal consistency of the subscales was assessed using Cronbach’s alpha coefficient. The adequacy of the model was assessed using the root mean square of residuals and Tucker Lewis Index.

Statistical analyses were performed using Excel (Microsoft® Excel® for Microsoft 365 MSO (version 2306 build 16.0.16529.20164, 64-bit), Redmond, WA, USA) and R version 4.3.1 (2023-06-16 ucrt) (Copyright© 2020, The R Foundation for Statistical Computing). The Psych and GPA rotation packages were used.

## 3. Results

Herein, 38 patients (All women, mean age: 68.7 ± 9.2 years, mean disease duration: 38.9 months, range 3–180) were enrolled. A total of 23.7% (9/38) patients had no underlying disease, whereas the remainder had comorbidities unrelated to oral pain and were taking multiple medications. Smoking habit accounted for 10.5% (4/38).

Table 1 summarizes the total scores of each scale. The SF-MPQ-2 (Original) showed floor effects. Cronbach’s alpha was 0.93 (0.88–0.96) and 0.83 (0.74–0.90) for the SF-MPQ-2 (original) and SF-MPQ-2 (Modified), respectively. Since Cronbach’s alpha was over 0.9, the SF-MPQ-2 (original) may have had redundant questions. The SF-MPQ-2 (Modified) and NRS assessment of pain demonstrated adequate reliability. Reportedly, the SF-MPQ-2 (Modified) had better reliability than the SF-MPQ-2 (Original). Fig. 2 shows scatter plots and line correlations between total scores of the NRS and SF-MPQ-2 (Original), NRS and SF-MPQ-2 (Modified), and SF-MPQ-2 (Original) and SF-MPQ-2 (Modified). The correlation coefficients were 0.15 (95% CI: −0.24, 0.50) (Fig. 2A), 0.76 (95% CI: 0.57, 0.87) (Fig. 2B), 0.23 (95% CI: −0.15, 0.55) (Fig. 2C), respectively. Only the correlation between the NRS and the SF-MPQ-2 (Modified) reached statistical significance.

**Table 1.** Descriptive statistics for total scores.

Total score	SF-MPQ-2 (Original)	SF-MPQ-2 (Modified)	NRS
Mean (SD)	13.8 (18.9)	6.5 (5.6)	3.0 (1.9)

*Abbreviations: SD: standard deviation; SF-MPQ-2: Short Form McGill Pain Questionnaire version 2; NRS: numerical rating scale.*

**Fig. 2.** Scatter plots and linear correlations between total scores. Total scores of (A) NRS and SF-MPQ-2 (Original), (B) NRS and SF-MPQ-2 (Modified), and (C) SF-MPQ-2 (Original) and SF-MPQ-2 (Modified). SF-MPQ-2: Short Form McGill Pain Questionnaire version 2; NRS: numerical rating scale.

Tables 2,3, and Fig. 3 shows the results of the factor analysis. Factor analysis was performed using a SF-MPQ-2 (Modified), demonstrating improved reliability and validity for BMS. The BMS-specific descriptors were extracted, and factor analysis was performed. The 8 descriptors (X3, X4, X6, X12, X14, X15, X17 and X18) were excluded owing to low selection rates (≤5 participants). Since items with high uniqueness contribute less to each factor, factors with uniqueness values of ≥0.70 were excluded. The following predictors were removed chronologically: X2, X7 and X8. The final factorial solution yielded three factors that accounted for 61.2% of the variance (Fig. 3). Descriptors with loadings ≥0.4 or ≥−0.4 were added to the subscales, with duplicate descriptors assigned to the subscale with the higher loading. Finally, X9, X10, X11 and X19; X5, X16, X20 and X21; and X1, X13 and X22 were loaded in Subscales 1, 2 and 3, respectively. The sampling adequacy of overall variables in the model was measured Kaiser-Meyer-Olkin, and the value was 0.68. The root mean square of the residuals was 0.07. Tucker Lewis Index of factoring reliability was 0.80. The results suggest that the model fit was not poor.

**Table 2.** Results of the correlation matrix.

Correlation matrix	X1	X5	X9	X10	X11	X13	X16	X19	X20	X21	X22
X1	1.00	0.12	0.24	0.24	0.31	−0.09	0.19	0.02	0.17	0.18	−0.32
X5	0.12	1.00	0.54	0.37	0.30	0.65	0.43	0.45	0.49	0.24	0.53
X9	0.24	0.54	1.00	0.64	0.36	0.56	0.28	0.66	0.37	0.57	0.41
X10	0.24	0.37	0.64	1.00	0.67	0.28	−0.05	0.79	−0.01	0.31	0.19
X11	0.31	0.30	0.36	0.67	1.00	0.25	−0.03	0.48	−0.16	0.13	0.18
X13	−0.09	0.65	0.56	0.28	0.25	1.00	0.20	0.46	0.48	0.50	0.73
X16	0.19	0.43	0.28	−0.05	−0.03	0.20	1.00	−0.11	0.27	0.36	0.11
X19	0.02	0.45	0.66	0.79	0.48	0.46	−0.11	1.00	0.23	0.26	0.32
X20	0.17	0.49	0.37	−0.01	−0.16	0.48	0.27	0.23	1.00	0.39	0.20
X21	0.18	0.24	0.57	0.31	0.13	0.50	0.36	0.26	0.39	1.00	0.30
X22	−0.32	0.53	0.41	0.19	0.18	0.73	0.11	0.32	0.20	0.30	1.00

**Table 3.** Results of the factor analysis.

Loadings:				
	Factor 1	Factor 2	Factor 3	Communality
X1	0.212	0.343	−0.550	0.465
X5	0.372	0.561	0.347	0.574
X9	0.634	0.534	0.142	0.707
X10	0.996	0	0	0.992
X11	0.675	0	0	0.456
X13	0.294	0.560	0.658	0.833
X16	0	0.547	0	0.299
X19	0.794	0.159	0.200	0.696
X20	0	0.720	0.107	0.530
X21	0.301	0.553	0.103	0.407
X22	0.215	0.251	0.804	0.756
	Factor 1	Factor 2	Factor 3	
SS loadings	2.892	2.243	1.591	
Proportion Var	0.263	0.204	0.145	
Cumulative	0.263	0.467	0.612	
Alpha reliability	0.860	0.669	0.120	

*Abbreviation: SS loadings: sums of squares of loadings; Var: variance.*

**Fig. 3.** Results of the factor analysis. (A) Scree plot; when three factors were used, the eigenvalues of the principal factors fell within an acceptable range (<0). (B) Factor model diagram; the relationship between latent factors and descriptors were shown in figure. FA: factor analysis; ML: maximum likelihood method’s factor.

We labeled each subscale as “nociceptive pain”, “neuropathic pain” and “affective pain”. Table 4 demonstrates Cronbach’s alpha reliability coefficients for each subscale.

**Table 4.** Cronbach’s alpha reliability coefficient for each subscale.

Total score/subscale score		Included items	Alpha reliability
SF-MPQ-2 (Modified)			
	Continuous pain descriptors	X1, X5, X6, X8, X9, and X10 (6 items)	0.618 (95% CI: 0.378, 0.787)
	Intermittent pain descriptors	X2, X3, X4, X11, X16, and X18 (6 items)	0.596 (95% CI: 0.342, 0.775)
	Neuropathic pain descriptors	X7, X17, X19, X20, X21, and X22 (6 items)	0.514 (95% CI: 0.209, 0.729)
	Affective descriptors	X12, X13, X14, and X15 (4 items)	0.752 (95% CI: 0.581, 0.864)
SF-MPQ-2 (Modified)			
	F1: nociceptive pain descriptors	X9, X10, X11, and X19 (4 items)	0.860 (95% CI: 0.762, 0.923)
	F2: neuropathic pain descriptors	X5, X16, X20, and X21 (4 items)	0.669 (95% CI: 0.441, 0.819)
	F3: affective pain descriptors	X1, X13, and X22 (3 items)	0.120 (95% CI: −0.554, 0.531)

*Abbreviation: SF-MPQ-2: Short Form McGill Pain Questionnaire version 2; F: Factor; CI: confidence interval.*

Fig. 4A shows the heat map of the existing subscales (continuous pain, intermittent pain, neuropathic pain, and affective descriptors). Fig. 4B shows a heat map illustrating the new subscales (nociceptive, neuropathic, and affective pain).

**Fig. 4.** Heatmap of existing subscales. (A) SF-MPQ-2 subscales (continuous pain, intermittent pain, neuropathic pain, affective descriptors). (B) New subscales (nociceptive pain, neuropathic pain, and affective pain).

## 4. Discussion

To our knowledge, this is the first study to demonstrate the reliability and validity of the SF-MPQ-2 for BMS. These analyses suggest that the SF-MPQ-2 has robust psychometric properties.

BMS is an intraoral burning sensation with no identifiable medical or dental cause. Patients with BMS are diagnosed by the exclusion of all other possible disorders, considering there are no specific laboratory tests (candidiasis, serum zinc, serum copper, serum iron, and serum vitamin B12) or tools for establishing a definitive diagnosis. In general, BMS predominantly affects middle-aged and older women (aged 50–70 years) [2, 5, 7], which is consistent with the age range of our participants. The manifestation of pain in BMS varies among patients, including “burning pain”, “tingling pain” and “numbness”. Although 7.89% (3/38) of patients had difficulty in qualitatively assessing pain on the SF-MPQ-2 (scoring 0 on all 22 items), SF-MPQ-2 was considered adequate for assessing pain in BMS.

Reportedly, BMS is associated with psychosocial problems and psychogenic factors, including anxiety, depression, and personality disorders [2, 3, 4, 5, 6, 7]. In this study, 15.8% (6/38) of patients had a history of psychiatric illness and were receiving treatment, primarily for depression or insomnia. Recent evidence suggests that “psychogenic” factors are not primary but secondary etiology of BMS pathophysiology [2, 3, 4, 5, 6, 7], with symptoms likely arising from neuropathic pain [2, 7]. At present, the diagnosis and treatment of BMS requires a comprehensive evaluation that includes psychosocial factors.

In the results of the four original subscales, several patients selected “continuous pain” and “neuropathic pain”, whereas fewer patients complained of “affective descriptors”. Although several studies have suggested an association between BMS and psychiatric disorders [3, 4, 5, 6, 7], our results suggest that the original subscales of the SF-MPQ-2 may not be appropriate for assessing BMS. Statistical analysis revealed low Cronbach’s alpha reliability for each original subscale of the SF-MPQ-2, and existing subscale methods showed poor performance in assessing BMS. Based on our factor analysis, the alpha reliability coefficients indicated that two factors had strong correlations among items within the factor, while the remaining factor had weak correlations among items within the factor. In other words, the two factors were grouped together with similar items. Therefore, 11 statistically significant items were selected and reclassified into three subscales. Moreover, the results of the scree plot suggest that the extraction of three factors is reasonable. Disjointed items are present in factors with low alpha reliability coefficients, allowing for the identification of subtypes (psychogenic). Of 11 items selected, only X13 was related to “affective descriptors”. “Affective pain” scores, including X13, were also low in the new subscale, and some participants had a strong association between BMS and mental state. Reportedly, Japanese individuals under-report pain intensity and emotional expression, suggesting possible ethnic influences on SF-MPQ-2 scores [20].

Conducting comprehensive interviews and maintaining effective communication with patients with BMS is vital. However, pain is complex in nature, disease duration is typically prolonged, and prolonged time in the dental chair remains a substantial challenge in current clinical practice. The time to complete the SF-MPQ-2 was approximately 5 min, although further simplification is needed for BMS treatment. A study investigating salivary biomarkers for BMS diagnosis and treatment monitoring is also underway. However, biomarker measurement is costly and time-consuming, highlighting the need for a simpler and more cost-effective assessment method. Considerably, we propose a novel assessment approach for BMS using an 11-item questionnaire with a four-point rating scale.

The Japanese version of the PainDETECT questionnaire was not suitable as a pain assessment tool for patients with BMS [21]. The PainDETECT questionnaire assesses nociceptive and neuropathic pain [21]. However, the PainDETECT questionnaire is constructed in a correlational way and uses cut-off scores to classify pain [21]. In other words, it may not be appropriate to perform factor analysis of nociceptive and neuropathic pain assessments with oblique rotation in the BMS pain assessment. Therefore, in this study, we ensured that nociceptive and neuropathic pain were evaluated independently by orthogonal rotation. Results of this study suggest that self-assessment scores for nociceptive and neuropathic pain may vary from patient to patient. Therefore, the nature of pain varies from patient to patient and treatment should be individualized.

This study has several limitations. The sample size was reduced because all cases with other pain triggers were excluded according to the ICOP diagnostic criteria. Therefore, this is an exploratory study, and further research with a larger sample size and multi-center design is needed to validate these preliminary findings. Herein, BMS was assessed using a self-administered questionnaire, which introduces potential subjective bias. In addition, cognitive function tests, such as the Hasegawa Dementia Scale-Revised (HDS-R) and Mini-Cog, were not administered. Alternatively, only patients who communicated adequately, responded to questionnaires without impairment, and had no observed cognitive dysfunction were selected for cognitive safety. Treatment history was not assessed in the study population. In some cases, treatment was already initiated during the survey. This study was a retrospective study, and the timing of data acquisition varied, so it was not possible to examine the treatment effect on each subscale. Therefore, in order to support the clinical practice of subscales, it is necessary to study the therapeutic effects of each subscale in prospective studies.

Several studies have proposed research diagnostic criteria and emphasized the notable role of psychological factors and psychological support in BMS. The evaluation of the therapeutic effect of each category is a subject of future research.

## 5. Conclusions

The SF-MPQ-2 is a valuable tool for patients with BMS. Although the SF-MPQ-2 should be considered as a tool for assessing BMS, its applicability in clinical settings requires more extensive study. The proposed classification system may offer improved treatment strategies and objective pain measurements should be used in future studies.

## Supplementary Material

Supplementary material associated with this article can be found, in the online version, at https://files.jofph.com/ files/article/1966379426072412160/attachment/ Supplementary%20material.pdf.


