# Supplementary material for: Exploration of a pain assessment tool on burning mouth syndrome
Source: J Oral Facial Pain Headache. 2025 Sep 12;39(3):183–90. doi: 10.22514/jofph.2025.060 (PMC12520451; doi:10.22514/jofph.2025.060)
Supplement: Supplementary file 1 [file Supplementary-material.pdf]

Short-Form McGill pain Questionnaire-2 (Original)

Date :   /   /   .

Patient Number : ____________ **Name :** ____________ **Age :** ______ **Sex : M /F**

This questionnaire provides you with a list of Words that describe some of the different qualities if pain and related symptoms. Please put an × through the numbers that best describe the intensity of each of the pain and related symptoms you felt during the past week. Use 0 if the word dose not describe your pai or related symptoms.

		None										Worst possible
X1	Throbbing pain	0	1	2	3	4	5	6	7	8	9	10
X2	Shooting pain	0	1	2	3	4	5	6	7	8	9	10
X3	Stabbing pain	0	1	2	3	4	5	6	7	8	9	10
X4	Sharp pain	0	1	2	3	4	5	6	7	8	9	10
X5	Cramping pain	0	1	2	3	4	5	6	7	8	9	10
X6	Gnawing pain	0	1	2	3	4	5	6	7	8	9	10
X7	Hot-burning pain	0	1	2	3	4	5	6	7	8	9	10
X8	Aching pain	0	1	2	3	4	5	6	7	8	9	10
X9	Heavy pain	0	1	2	3	4	5	6	7	8	9	10
X10	Tender	0	1	2	3	4	5	6	7	8	9	10
X11	Splitting pain	0	1	2	3	4	5	6	7	8	9	10
X12	Tiring-exhausting	0	1	2	3	4	5	6	7	8	9	10
X13	Sickening	0	1	2	3	4	5	6	7	8	9	10
X14	Fearful	0	1	2	3	4	5	6	7	8	9	10
X15	Punishing-cruel	0	1	2	3	4	5	6	7	8	9	10
X16	Electric-shock pain	0	1	2	3	4	5	6	7	8	9	10
X17	Cold-freezing pain	0	1	2	3	4	5	6	7	8	9	10
X18	Piercing	0	1	2	3	4	5	6	7	8	9	10
X19	Pain caused by light touch	0	1	2	3	4	5	6	7	8	9	10
X20	Itching	0	1	2	3	4	5	6	7	8	9	10
X21	Tingling or pins and needles	0	1	2	3	4	5	6	7	8	9	10
X22	Numbness	0	1	2	3	4	5	6	7	8	9	10

Numerical Rating Scale : ______/ 10

Short-Form McGill pain Questionnaire-2 (Modified)

Date : / / .

Patient Number : **Name :** **Age :** **Sex : M /F**

This questionnaire provides you with a list of Words that describe some of the different qualities if pain and related symptoms. Please put an × through the numbers that best describe the intensity of each of the pain and related symptoms you felt during the past week. Use 0 if the word dose not describe your pai or related symptoms.

		None			Worst possible
X1	Throbbing pain	0	1	2	3
X2	Shooting pain	0	1	2	3
X3	Stabbing pain	0	1	2	3
X4	Sharp pain	0	1	2	3
X5	Cramping pain	0	1	2	3
X6	Gnawing pain	0	1	2	3
X7	Hot-burning pain	0	1	2	3
X8	Aching pain	0	1	2	3
X9	Heavy pain	0	1	2	3
X10	Tender	0	1	2	3
X11	Splitting pain	0	1	2	3
X12	Tiring-exhausting	0	1	2	3
X13	Sickening	0	1	2	3
X14	Fearful	0	1	2	3
X15	Punishing-cruel	0	1	2	3
X16	Electric-shock pain	0	1	2	3
X17	Cold-freezing pain	0	1	2	3
X18	Piercing	0	1	2	3
X19	Pain caused by light touch	0	1	2	3
X20	Itching	0	1	2	3
X21	Tingling or pins and needles	0	1	2	3
X22	Numbness	0	1	2	3

Numerical Rating Scale : ______/ 10
